# Effects of guanidinoacetic acid supplementation on liver and breast muscle fat deposition, lipid levels, and lipid metabolism-related gene expression in ducks

**DOI:** 10.3389/fvets.2024.1364815

**Published:** 2024-02-16

**Authors:** Hongzhi Wu, Jiajun Xie, Weiqi Peng, Fengjie Ji, Jinyu Qian, Qian Shen, Guanyu Hou

**Affiliations:** ^1^Tropical Crop Genetic Resource Research Institute, Chinese Academy of Tropical Agricultural Sciences, Haikou, China; ^2^College of Animal Science and Technology, Northeast Agricultural University, Harbin, China; ^3^Hainan Xuhuai Technology Co., Ltd., Haikou, China

**Keywords:** guanidinoacetic acid, fat deposition, lipase activity, fatty acid composition, lipid metabolism genes

## Abstract

Exogenous supplementation of guanidinoacetic acid can mechanistically regulate the energy distribution in muscle cells. This study aimed to investigate the effects of guanidinoacetic acid supplementation on liver and breast muscle fat deposition, lipid levels, and lipid metabolism-related gene expression in ducks. We randomly divided 480 42 days-old female Jiaji ducks into four groups with six replicates and 20 ducks for each replicate. The control group was fed the basal diet, and the experimental groups were fed the basal diet with 400, 600, and 800 mg/kg (GA400, GA600, and GA800) guanidinoacetic acid, respectively. Compared with the control group, (1) the total cholesterol (*p* = 0.0262), triglycerides (*p* = 0.0357), malondialdehyde (*p* = 0.0452) contents were lower in GA400, GA600 and GA800 in the liver; (2) the total cholesterol (*p* = 0.0365), triglycerides (*p* = 0.0459), and malondialdehyde (*p* = 0.0326) contents in breast muscle were decreased in GA400, GA600 and GA800; (3) the high density lipoprotein (*p* = 0.0356) and apolipoprotein-A1 (*p* = 0.0125) contents were increased in GA600 in the liver; (4) the apolipoprotein-A1 contents (*p* = 0.0489) in breast muscle were higher in GA600 and GA800; (5) the lipoprotein lipase contents (*p* = 0.0325) in the liver were higher in GA600 and GA800; (6) the malate dehydrogenase contents (*p* = 0.0269) in breast muscle were lower in GA400, GA600, and GA800; (7) the *insulin induced gene 1* (*p* = 0.0326), *fatty acid transport protein 1* (*p* = 0.0412), and *lipoprotein lipase* (*p* = 0.0235) relative expression were higher in GA400, GA600, and GA800 in the liver; (8) the *insulin induced gene 1* (*p* = 0.0269), *fatty acid transport protein 1* (*p* = 0.0234), and *lipoprotein lipase* (*p* = 0.0425) relative expression were increased in GA400, GA600, and GA800 in breast muscle. In this study, the optimum dosage of 600 mg/kg guanidinoacetic acid improved the liver and breast muscle fat deposition, lipid levels, and lipid metabolism-related gene expression in ducks.

## 1 Introduction

Lipids are essential for body energy storage and supply and are also important structural components of biofilms (1, 2). The biochemical process of lipid metabolism after absorption is mainly divided into the metabolism of triglycerides, phospholipids, cholesterol, and plasma lipoproteins in animals (2). This process is primarily regulated by hormones, diet nutrition, and biochemical enzyme activity in a complex and precise way, and it is finally transformed into the material components required for various acceptable biochemical reactions (2, 3). Lipid metabolism in animals is a complex biochemical process, which is regulated by various lipases and related genes, such as *free fatty acid receptor 4* (*FFAR4*), *fatty acid transport protein 1* (*FATP1*), *insulin induced gene 1* (*INSIGI1*), *lipoprotein lipase* (*LPL*), *stearoyl-CoA desaturase* (*SCD*), *fatty acid synthase* (*FAS*) and so on (2, 4). Guanidinoacetic acid is a self-existing substance in animals that is a precursor of creatine and plays a vital role in creatine biosynthesis (5, 6). The additional addition of guanidinoacetic acid causes the body to produce a large amount of phosphate group transfer substance, phosphocreatine, which in turn provides energy for the efficient work of tissues such as muscles, brain, and gonads and promotes the continuous distribution of nutrients to the muscles and other tissues, thus changes the animal size (7, 8). Exogenous supplementation of guanidinoacetic acid can mechanistically regulate the energy distribution of muscle cells (9, 10), thus more effectively reducing the activity of related key enzymes through multiple metabolic pathways, inhibiting fatty acid synthesis in the liver, promoting fatty acid β-oxidation, and ultimately reducing the deposition of abdominal fat in broiler chickens by reducing the size or number of abdominal adipocytes (11–13); at the same time, exogenous supplementation of guanidinoacetic acid reduces the consumption of feed-added arginine, so that this part of the amino acid from another pathway, such as through the activation of the Adenosine 5′-monophosphate (AMP)-activated protein kinase signaling pathway, to promote myocyte membrane glucose transporter 4 receptor expression, improve myocyte glucose uptake, which in turn promotes muscle growth and intramuscular fat deposition, and thus improve the quality of meat texture and flavor (14, 15).

Research on guanidinoacetic acid in livestock production has focused on the growth performance and meat quality of pigs, chickens, and ruminants. Sharma et al. found that guanidinoacetic acid improved the abdominal fat yields and breast meat creatine concentration in broilers (16). Majdeddin et al. (17) reported that the guanidinoacetic acid improved the feed conservation and meat quality in broilers. He et al. (18) found that guanidinoacetic acid maximized the growth performance of growing-finishing pigs. Jayaraman et al. (19) reported that guanidinoacetic acid improved average daily gain, feed efficiency, and lean meat yield and reduced back-fat thickness in pigs. Zhang et al. (20) found that the guanidinoacetic acid improved the creatine concentration in Kazakh male lambs. There is minimal research on waterfowl, especially on the fat deposition and distribution in ducks. Therefore, the purpose of this study was to investigate the effects of guanidinoacetic acid on the liver and breast muscle fat deposition, lipid levels, and lipid metabolism-related gene expression in ducks to provide more theoretical bases for their application in the diet of ducks and to contribute to the sustainable and healthy development of waterfowl farming.

## 2 Materials and methods

All procedures involving care and management were approved by the Institutional Animal Care and Use Committee of the Chinese Academy of Tropical Agricultural Sciences (Approval No.: CATAS-20220819-2).

### 2.1 Animals and experimental design

We randomly divided 480 42 days-old female Jiaji ducks, with 935 ± 5.00 g, into four groups with six replicates and 20 ducks for each replicate. The control group (CG) was fed the lab-formulated basal diet (corn-soybean diets), and the experimental groups were fed the control diet supplemented with 400, 600, and 800 mg/kg guanidinoacetic acid in the form of powder (GA400, GA600, and GA800, respectively). Furthermore, the guanidinoacetic acid, with a concentration ≥99.00%, was bought from Shandong Yatou Biotechnology Co. Ltd., Jinan, China. The composition of the basal diets and nutritional levels are shown in Table 1. The soybean meal (42.00% crude protein, irregular fragment) and peanut meal (47.80% crude protein, irregular fragment) were broken into powder using a grinder (MJW-W Mechanical crusher, Qingdao EPIC Powder Machinery, Qingdao, China), and the mixed with the corn (powder) and other ingredients in a blender (V-200, Wuxi Zhuangcheng Equipment Technology Co., Ltd., Wuxi, China). Finally, put the mixed feed into a double waterproof and moisture-proof feed bag and store it for later use. Feed samples were randomly taken from each treated feed and used to detect the guanidinoacetic acid contents. Before the trial, the duck house was airtightly disinfected using a mixture of formalin and potassium permanganate for 3 days and ventilated. All ducks were reared on a net bed. The light time, humidity, and temperature of the duck house were controlled at 18 h, 70%, and 26°C for 48 days. Use 12-watt LED tubes to adjust the duck house light time requirements. Depending on the growth of ducks, it is decided whether to add extra multiple vitamins and multiple minerals to their drinking water.

**Table 1:** Composition and nutrient levels of corn-soybean diets, air-dry basis.

Items	Contents	Nutrient levels	Contents
Corn, %	60.20	Metabolizable energy^c^, MJ/kg	11.30
Soybean meal (42.00%), %	20.50	Crude protein^d^, %	16.00
Peanut meal (47.80), %	16.14	Lysine^d^, %	0.90
Calcium hydrogen phosphate, %	1.20	Methionine^d^, %	0.42
Limestone, %	1.00	Calcium^d^, %	0.70
Multiple vitamin premix^a^, %	0.03	Available phosphorus^d^, %	0.35
Multiple mineral premix^b^, %	0.10		
Choline chloride, %	0.06		
Sodium chloride, %	0.30		
Lysine, %	0.17		
Methionine, %	0.20		
Mildew inhibitors, %	0.10		
Total	100.00		

a Multiple vitamin premix per kilogram contains VA 50,000,000 IU, VB_1_ 10,000 mg, VB_2_ 20,000 mg, VB_6_ 10,000 mg, VB_12_ 5,000 mg, VC 4,000 mg, VD 1,000,000 IU, VE 60,000 IU, VK_3_ 8,000 mg, folic acid 2,500 mg, niacin 800 mg, pantothenic acid 300 mg, lysine 800 mg, methionine 550 mg, tryptophan 200 mg, biotin 220 mg.

b Multiple mineral premix per kilogram contains Cu as copper sulfate 5.00 mg, Fe as ferrous sulfate 50.00 mg, Zn as zinc sulfate 55.00 mg, Mn as manganese sulfate 55.00 mg, I as potassium iodide 0.30 mg, Se as sodium selenite 0.22 mg.

c Nutritional levels are calculated.

d Analysed content.

### 2.2 Sample collections

One duck with average body weight in each replicate was selected to slaughter after 12 h of fasting to collect the data and samples at the 90 days of age. Determine the abdominal fat percentage of ducks according to NY/T823-2004 standard. Ten grams of the liver and breast muscles in the same part of the ducks were collected and stored at −20°C to detect the biochemistry parameters. About three portions of 5 g liver and breast muscle were collected and stored at −80°C to evaluate the relative expression of genes related to lipid metabolism.

### 2.3 Sample biochemical analysis

The liver and breast muscles were homogenated with the E6618 TissueMaster^™^ High-Throughput Tissue Homogenizer, with 1.5/2 mL × 48 Adaptor, Shanghai, China. The cholesterol, triglycerides, phospholipid, malondialdehyde, intramural fat, creatine, high density lipoprotein, low density lipoprotein, apolipoprotein-A1, apolipoprotein-B, lipoprotein lipase, total lipase, and malate dehydrogenase were all determined by the HITACHI Automatic Analyzer 3,500 (HITACHI, Ibaraki-Ken, Japan). The reagent kits used in this trial were purchased from the Nanjing Jiancheng Biotechnology Co., Ltd., Jiangsu, China, and the procedures were carried out strictly following the manufacturer’s instructions. In breast muscle, the fatty acid composition was determined using gas chromatography with TRACE^™^ 1310E Gas chromatograph (1310E, Shanghai, China). The fatty acids were extracted by a mixture of benzene and petroleum ether (1,1) and rapidly methylated. The content of fatty acids was quantitatively calculated by the normalization method of peak area (21). After extracting the muscle with petroleum ether, the intramuscular fat was obtained by steaming the solvent. The intramuscular fat content was calculated after extraction with petroleum ether using an automatic fat extraction instrument (XD-SXT-210, Shanghai, China) (22).

### 2.4 Lipid metabolism-related gene expression

Following the RNA extraction method, total RNA was isolated from the liver, breast and leg muscles with TRIzol (Sigma, Saint Louis, MO, United States). A 2% agarose gel electrophoresis was performed to assess the quality of the RNA, and total RNA concentration and purity (A260/A280 ratio, 1.80–2.00 is the qualification standard) were ascertained with an ultra-micro spectrophotometer, NanoPhotometer, Implen, German. The primer sequences are shown in Table 2, and primer sequences for duck genes were designed and synthesized by Sangon Biotech, Shanghai, China. All of the process was conducted under RNase-free condition. cDNA was synthesized from RNA using oligo (dT) primers (Invitrogen) in a 40 μL reaction mixture by using a SuperScript III kit (Invitrogen) according to the manufacturer’s instructions. ABI PRISM 7500 SDS thermal cycler (Applied Biosystems, Foster City, CA, United States) was used to determine samples, followed by one cycle at 95°C for 30 s and 40 cycles of 95°C for 5 s and 60°C for 34 s. Based on the 2^−∆∆Ct^ method, relative gene mRNA expressions were normalized by glyceraldehyde-3-phosphate dehydrogenase expression, respectively (23). All of the processes were performed in triplicates under RNase-free conditions. RT-qPCR products were cloned into a pMD18-T vector (TaKaRa) and sequenced by the Sanger method. The sequencing results were compared with the gene sequences in NCBI, and the genes amplified by the primers in Table 2 were verified as the target genes according to the alignment sequence results (23, 24).

**Table 2:** Primers used for quantitative real-time RT-qPCR.

Gene	Gene name	Forward and reverse primers	Product size	Accession No.
SCD	Stearoyl CoA desaturase	F: 5′-GTTCTGTGTTTCGTGGTGAG-3′	351	XM_027460089.2
		R: 5′-CGAGGGCTTGTAGTATTTCC-3′		
INSIG1	Insulin induced gene 1	F: 5′-CTCTGGTGGACATTTGATCG-3′	149	XM_005026017.1
		R: 5′-GGAACCAAGAACGGATGTA-3′		
FATP1	Fatty acid transport protein 1	F: 5′-TCGTGGGGCAGATAAATCAACA-3′	242	XM_005018222.4
		R: 5′-GTGACTCAACATCCCTTCCACT-3′		
LPL	Lipoprotein lipase	F: 5′-AGTACGCTGATGCCCTTACG-3′	191	FJ185781.1
		R: 5′-AGCAATCAGACGCAGAGCTT-3′		
FAS	Fatty acid synthetase	F: 5′-TGGAACCCTACTAAGCAGCC-3′	110	XM_027459847.2
		R: 5′-AAGATTGTCCGCCTTCCTGA-3′		
FFAR4	Free fatty acid receptor 4	F: 5′-GCACCGTCATCATCCTCTC-3′	155	XM_005013601.1
		R: 5′-GGCATAGGTCACATCCCAAA-3′		
GADPH	Glyceraldehyde-3-phosphate dehydrogenase	F: 5′-ACATGGCATCCAAGGAGTGAG-3′	144	XM_038180584.1
		R: 5′-GGGGAGACAGAAGGGAACAGA-3′		

### 2.5 Statistical analysis of data

Statistical analyses were conducted using SAS 9.4. Data were expressed as mean ± standard error of the mean. Different treatments were statistically compared using one-way ANOVA or Welch ANOVA after the Kolmogorov–Smirnov. The Barteet’s test was used for data that meets the normal distribution, otherwise the Levene’s test was used. Statistical differences among groups were assessed using Duncan’s multiple range test. The Dunnett’ T3 test was used when Welch ANOVA was employed. All experimental data in this study meet the requirement of normal distribution. The test results of all analyses were considered significant at *p* < 0.05.

## 3 Results

### 3.1 Effects of guanidinoacetic acid on lipid levels in the liver and breast muscle

**Figure 1:**
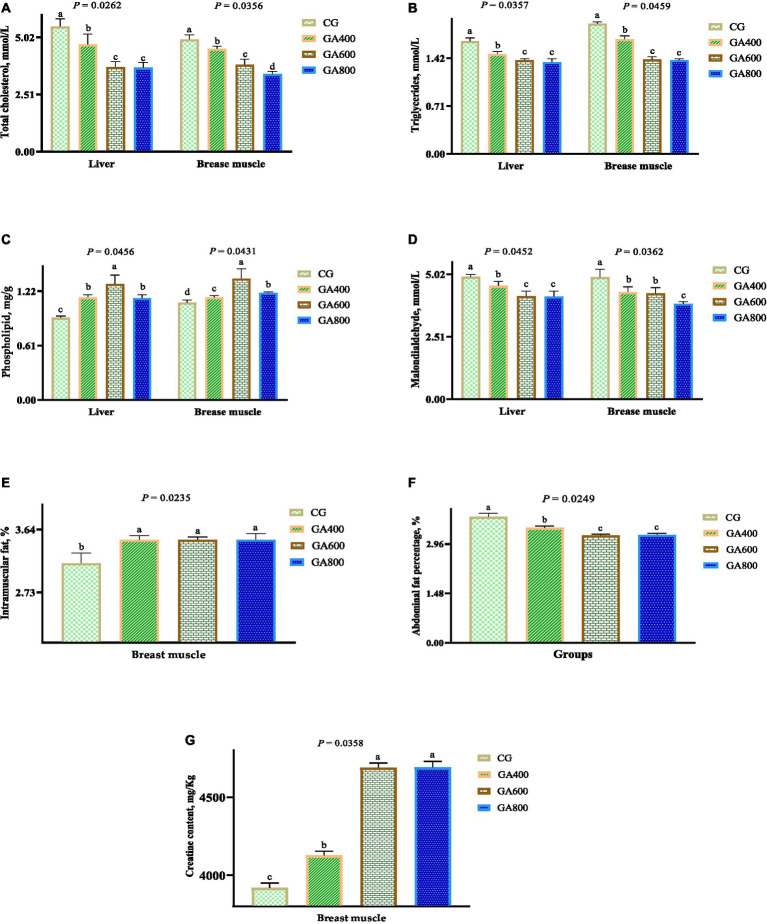
Effects of guanidinoacetic acid on lipid levels in ducks. The data in CG, GA400, GA600 and GA800 were **(A)** total cholesterol (mmol/L) in the liver 5.49 ± 0.34^a^, 4.70 ± 0.46^b^, 3.71 ± 0.24^c^, 3.68 ± 0.23^c^, respectively, *p* = 0.0262; and in breast muscle 4.92 ± 0.20^a^, 4.51 ± 0.12^b^, 3.81 ± 0.25^c^, 3.40 ± 0.12^d^, respectively, *p* = 0.0365; **(B)** triglycerides (mmol/L) in the liver 1.67 ± 0.05^a^, 1.48 ± 0.04^b^, 1.39 ± 0.02^c^, 1.36 ± 0.05^c^, respectively, *p* = 0.0357; and in breast muscle 1.93 ± 0.03^a^, 1.70 ± 0.05^b^, 1.40 ± 0.04^c^, 1.39 ± 0.02^c^, respectively, *p* = 0.0459; **(C)** phospholipid (mg/g) in the liver 0.92 ± 0.02^c^, 1.15 ± 0.03^b^, 1.30 ± 0.10^a^, 1.14 ± 0.04^b^, respectively, *P* = 0.0456; and in breast 1.09 ± 0.03^d^, 1.15 ± 0.02^c^, 1.36 ± 0.11^a^, 1.20 ± 0.01^b^, respectively, *p* = 0.0431; **(D)** malondialdehyde (mmol/L) in the liver 4.91 ± 0.10^a^, 4.55 ± 0.18^b^, 4.13 ± 0.21^c^, 4.12 ± 0.22^c^, respectively, *p* = 0.0452; in breast muscle 4.90 ± 0.32^a^, 4.30 ± 0.21^b^, 4.25 ± 0.23^b^, 3.83 ± 0.09^c^, respectively, *p* = 0.0326; **(E)** intramuscular fat (%) in breast muscle 3.15 ± 0.15^b^, 3.49 ± 0.06^a^, 3.49 ± 0.04^a^, 3.49 ± 0.09^a^, respectively, *p* = 0.0235; **(F)** abdominal fat percentage (%) of ducks 3.77 ± 0.11^a^, 3.44 ± 0.06^b^, 3.21 ± 0.04^c^, 3.23 ± 0.05^c^, respectively, *p* = 0.0249; **(G)** creatine contents in breast muscle 3,920 ± 30.59^c^, 4,127 ± 26.85^b^, 4,689 ± 30.24^a^, 4,691 ± 38.67^a^, respectively, *p* = 0.0358.

In liver, the total cholesterol contents (*p* = 0.0262), triglycerides contents (*p* = 0.0357), and malondialdehyde contents (*p* = 0.0452) were lower in GA400, GA600, and GA800 than in the control group. The phospholipid contents (*p* = 0.0456) in the liver were increased by 25.00, 41.30, and 23.91%, respectively, in GA400, GA600, and GA800 compared with the control group (Figures 1A–D).

In breast muscle, the intramuscular fat contents (*p* = 0.0235) and phospholipid contents (*p* = 0.0431) were higher in GA400, GA600, and GA800 than in the control group. The total cholesterol (*p* = 0.0365), triglycerides (*p* = 0.0459), and malondialdehyde (*p* = 0.0326) contents in breast muscle were decreased in GA400, GA600, and GA800 compared with the control group (Figures 1A–E).

The abdominal fat percentage (*p* = 0.0249) of ducks was 8.75, 14.85, and 14.32%, respectively, lower in GA400, GA600, and GA800 than in the control group (Figure 1F).

The creatine contents (*p* = 0.0358) in breast muscle were 5.28, 19.62, and 19.67%, respectively, higher in GA400, GA600, and GA800 than in the control group (Figure 1G).

### 3.2 Effects of guanidinoacetic acid on lipoprotein levels in the liver and breast muscle

In the liver, the high density lipoprotein contents (*p* = 0.0356) and apolipoprotein-A1 contents (*p* = 0.0125) were increased by 16.32, 13.92, and 13.92% vs. 33.33, 23.08, and 33.33%, respectively, in GA600 compared with the CG, GA400, and GA800. The low density lipoprotein contents (*p* = 0.0419) in the liver were lower in GA600 and GA800 than in the control group. The apolipoprotein-B contents (*p* = 0.0256) were lower in GA400, GA600, and GA800 than in the control group (Table 3).

**Table 3:** Effects of guanidinoacetic acid on lipoprotein levels in ducks.

Items	CG	GA400	GA600	GA800	*p*-value
*Liver*					
HDL, mmol/L	1.90 ± 0.03^b^	1.94 ± 0.01^b^	1.94 ± 0.01^b^	2.21 ± 0.02^a^	0.0356
LDL, mmol/L	2.60 ± 0.12^a^	2.61 ± 0.12^a^	1.71 ± 0.10^b^	1.44 ± 0.12^c^	0.0419
Apo-A1, g/L	0.12 ± 0.01^b^	0.13 ± 0.01^b^	0.16 ± 0.01^a^	0.12 ± 0.01^b^	0.0125
Apo-B, g/L	0.85 ± 0.03^a^	0.80 ± 0.01^b^	0.77 ± 0.01^c^	0.73 ± 0.01^d^	0.0256
*Breast muscles*					
HDL, mmol/L	1.62 ± 0.03^d^	1.70 ± 0.02^c^	1.92 ± 0.02^b^	2.13 ± 0.03^a^	0.0251
LDL, mmol/L	1.76 ± 0.11^a^	1.54 ± 0.03^b^	1.35 ± 0.03^c^	1.34 ± 0.01^c^	0.0357
Apo-A1, g/L	0.15 ± 0.01^c^	0.15 ± 0.01^c^	0.19 ± 0.01^b^	0.22 ± 0.01^a^	0.0489
Apo-B, g/L	0.75 ± 0.01^a^	0.66 ± 0.01^b^	0.66 ± 0.01^b^	0.60 ± 0.01^c^	0.0457

^a,b,c,d^Means within a row with no common superscripts differ significantly (*p* < 0.05). HDL, high density lipoprotein; LDL, low density lipoprotein; Apo-A1, apolipoprotein-A1; Apo-B, apolipoprotein-B.

The high density lipoprotein contents (*p* = 0.0251) in breast muscle were higher in GA400, GA600, and GA800 than in the control group. The low density lipoprotein contents (*p* = 0.0357) and apolipoprotein-B contents (*p* = 0.0457) in breast muscle were decreased in GA400, GA600, and GA800 compared with the control group. The apolipoprotein-A1 contents (*p* = 0.0489) in breast muscle were 26.67 and 46.67%, respectively, higher in GA600 and GA800 than in the control group (Table 3).

### 3.3 Effects of guanidinoacetic acid on enzymatic activity in the liver and breast muscle

The lipoprotein lipase contents (*p* = 0.0325) in the liver were 34.05 and 47.03%, respectively, higher in GA600 and GA800 than in the control group. The total lipase contents (*p* = 0.0256) in the liver were increased in GA400, GA600, and GA800 compared with the control group. The malate dehydrogenase contents (*p* = 0.0443) in the liver were 30.01, 45.93, and 46.76%, respectively, lower in GA400, GA600, and GA800 than in the control group (Table 4).

**Table 4:** Effects of guanidinoacetic acid on enzymatic activity in ducks.

Items	CG	GA400	GA600	GA800	*p*-value
*Liver*					
Lipoprotein lipase, μmolFFA/mL h	1.85 ± 0.03^c^	1.89 ± 0.02^c^	2.48 ± 0.14^b^	2.72 ± 0.08^a^	0.0325
Total lipase, U/mg protein	10.56 ± 0.23^c^	12.10 ± 0.12^b^	14.26 ± 0.35^a^	14.32 ± 0.49^a^	0.0256
Malate dehydrogenase, U/mL	56.69 ± 10.33a	39.68 ± 2.26b	30.65 ± 2.07c	30.18 ± 6.06^c^	0.0443
*Breast muscles*					
Lipoprotein lipase, μmolFFA/mL h	1.96 ± 0.01^c^	2.01 ± 0.03^b^	2.34 ± 0.05^a^	2.37 ± 0.11^a^	0.0368
Total lipase, U/mg protein	8.12 ± 0.21^b^	8.10 ± 0.32^b^	9.56 ± 0.26^a^	9.62 ± 0.29^a^	0.0462
Malate dehydrogenase, U/mL	46.76 ± 3.73^a^	40.69 ± 2.26^b^	40.91 ± 2.10^b^	40.96 ± 2.92^b^	0.0269

^a,b,c^Means within a row with no common superscripts differ significantly (*p* < 0.05).

In breast muscle, the lipoprotein lipase contents (*p* = 0.0368) were 2.55, 19.39, and 20.92%, respectively, higher in GA400, GA600, and GA800 than in the control group. The total lipase contents (*p* = 0.0462) in breast muscle were increased in GA600 and GA800 compared with the control group. The malate dehydrogenase contents (*p* = 0.0269) in breast muscle were lower in GA400, GA600, and GA800 than in the control group (Table 4).

### 3.4 Effects of guanidinoacetic acid on fatty acid composition in breast muscle

Dietary guanidinoacetic acid supplementation did not influence the saturated fatty acid (SFA) contents (*p* = 0.6752), monounsaturated fatty acid (MUFA) contents (*p* = 0.0910), and polyunsaturated fatty acid (PUFA) contents (*p* = 0.1257) among groups (Table 5).

**Table 5:** Effects of guanidinoacetic acid on breast muscle fatty acid proportions (%) in ducks.

Items	CG	GA400	GA600	GA800	*p*-value
SFA	56.92 ± 1.26	55.24 ± 1.34	56.68 ± 1.29	57.26 ± 1.27	0.6752
MUFA	25.42 ± 1.21	27.16 ± 1.26	25.46 ± 1.20	24.35 ± 1.59	0.0910
PUFA	17.66 ± 1.06	17.60 ± 0.98	17.86 ± 1.20	18.39 ± 1.59	0.1257

SFA, saturated fatty acid; MUFA, monounsaturated fatty acid; PUFA, polyunsaturated fatty acid.

### 3.5 Effects of guanidinoacetic acid on lipid metabolism-related gene expression in the liver and breast muscle in ducks

**Figure 2:**
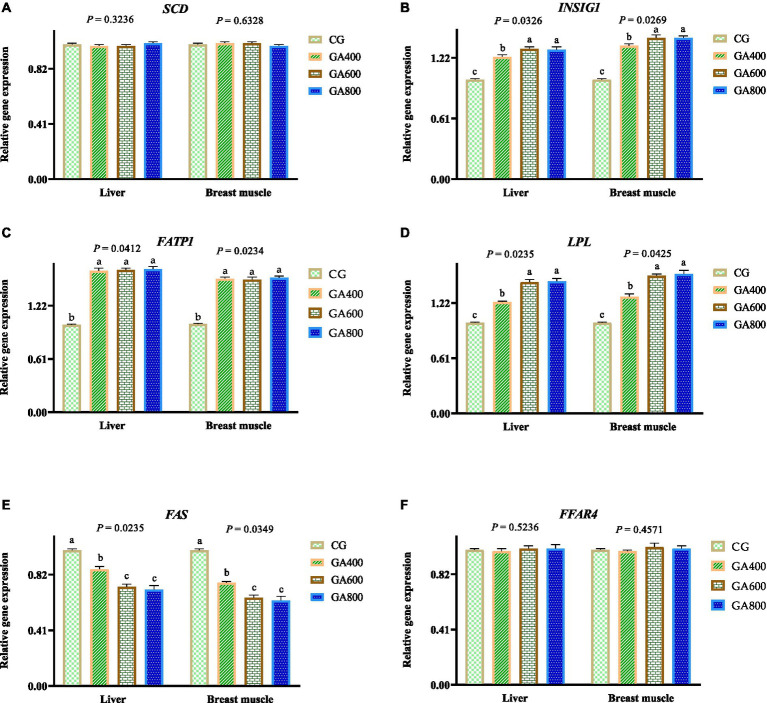
Effects of guanidinoacetic acid on lipid metabolism-related gene expression in ducks. The data in CG, GA400, GA600, and GA800 were **(A)** *SCD* (*stearoyl CoA desaturase*) in the liver 1.00 ± 0.01, 0.99 ± 0.01; 0.99 ± 0.01, 1.01 ± 0.01, respectively, *p* = 0.3236; and in breast muscle 1.00 ± 0.01, 1.01 ± 0.01, 1.01 ± 0.01, 0.99 ± 0.01, respectively, *p* = 0.6328; **(B)** *INSIG1* (*insulin induced gene 1*) in the liver 1.00 ± 0.01^c^, 1.23 ± 0.02^b^, 1.31 ± 0.02^a^, 1.3 ± 0.03^a^, respectively, *p* = 0.0326; and in breast muscle 1 ± 0.01^c^, 1.34 ± 0.02^b^, 1.42 ± 0.03^a^, 1.42 ± 0.02^a^, respectively, *p* = 0.0269; **(C)** *FATP1* (*fatty acid transport protein 1*) in the liver 1 ± 0.01^b^, 1.62 ± 0.03^a^, 1.63 ± 0.02^a^, 1.64 ± 0.03^a^, respectively, *p* = 0.0412; and in breast muscle 1.00 ± 0.01^b^, 1.53 ± 0.02^a^, 1.52 ± 0.03^a^, 1.54 ± 0.02^a^, respectively, *p* = 0.0234; **(D)** *LPL* (*lipoprotein lipase*) in the liver 1.00 ± 0.01^c^, 1.23 ± 0.01^b^, 1.45 ± 0.03^a^, 1.46 ± 0.03^a^, respectively, *p* = 0.0235; and in breast muscle 1 ± 0.01^c^, 1.29 ± 0.03^b^, 1.52 ± 0.02^a^, 1.54 ± 0.04^a^, respectively, *p* = 0.0425; **(E)** *FAS* (*fatty acid synthetase*) in the liver 1.00 ± 0.01^a^, 0.86 ± 0.02^b^, 0.73 ± 0.02^c^, 0.71 ± 0.03^c^, respectively, *p* = 0.0235; and in breast muscle 1.00 ± 0.01^a^, 0.76 ± 0.01^b^, 0.65 ± 0.02^c^, 0.63 ± 0.03^c^, respectively, *p* = 0.0349; **(F)** *FFAR4* (*free fatty acid receptor 4*) in the liver 1 ± 0.01, 0.99 ± 0.02, 1.01 ± 0.02, 1.01 ± 0.03, respectively, *p* = 0.5236; and in 1.00 ± 0.01, 0.99 ± 0.01, 1.02 ± 0.03, 1.01 ± 0.02, respectively, *p* = 0.4571.

In the liver, the relative *insulin induced gene 1* expressions (*p* = 0.0326), *fatty acid transport protein 1* expressions (*p* = 0.0412), and *Lipoprotein lipase* expressions (*p* = 0.0235) were 23.00, 31.00, and 30.00% vs. 62.00, 63.00, and 64.00% vs. 23.00, 45.00, and 46.00%, respectively, higher in GA400, GA600, and GA800 than in the control group. The *fatty acid synthetase* relative expression (*p* = 0.0235) was decreased by 14.00, 27.00, and 29.00%, respectively, in GA400, GA600, and GA800 compared with the control group (Figure 2).

In breast muscle, the relative *insulin induced gene 1* expressions (*p* = 0.0269), *Fatty acid transport protein 1* expressions (*p* = 0.0234), and *Lipoprotein lipase* relative expressions (*p* = 0.0425) were increased by 34.00, 42.00, and 42.00% vs. 53.00, 52.00, and 54.00% vs. 29.00, 52.00, and 54.00%, respectively, in GA400, GA600, and GA800 compared with the control group. The *fatty acid synthetase* relative expression (*p* = 0.0349) was 24.00, 35.00, and 37.00%, respectively, lower in GA400, GA600, and GA800 than in the control group. (Figure 2).

## 4 Discussion

Fat cells of female animals have more mitochondria, which can better metabolize fat and other substances (25). Therefore, female ducks were selected as the research object in this study. Almost all organs and tissues of poultry can synthesize cholesterol, of which 70–80% of cholesterol is synthesized in liver tissue (26–28). Cholesterol is an essential molecule in animals, and its metabolism plays a vital role in animal health (27). Triglyceride is an organic compound produced by esterification of three hydroxyl groups in glycerol and three fatty acid molecules (29). As a non-polar substance, triglyceride is stored in a non-hydrated form and is the energy substance with the largest reserve and production capacity in the animal (30, 31). Phospholipids are lipid compounds containing phospholipid roots consisting of lecithin, inositol phospholipids, and ceruloplasmin (32). Phospholipids play a significant role in activating cells, maintaining primary and primary metabolism and hormone secretion, and enhancing immunity and regeneration (9). In addition, phospholipids promote fat metabolism, lower serum cholesterol, and improve blood circulation (32, 33). Malondialdehyde is the end product of lipid peroxidation, and its production exacerbates cell membrane damage (34, 35). Sharma et al. (16) found that a high dose of guanidinoacetic acid made up for the arginine deficiency in the diet, and increased the contents of intermuscular fat, abdominal fat, and creatine in the breast muscle of broilers. Majdeddin et al. (17) reported that the guanidinoacetic acid improved the muscle creatine concentration in broilers. Majdeddin et al. (9) found that the increased the muscle creatine contents to enhance the energy metabolism in Ross 308 broilers. The cholesterol, malondialdehyde, and triglycerides are products of lipid metabolism (1). In this study, the total cholesterol, malondialdehyde, triglycerides, and abdominal fat percentage were decreased, and the phospholipid, intramuscular fat, and creatine contents were increased in liver and breast muscles in the groups treated with guanidinoacetic acid, especially in GA600, indicating that the guanidinoacetic acid improved the lipid metabolism in ducks, changed the fat deposition and lipid levels in liver and breast muscles.

High density lipoprotein is rich in proteins such as apolipoprotein-A1 and lecithin-cholesterol acyltransferase, and lecithin-cholesterol acyltransferase can be activated by apolipoprotein-A1 in high density lipoprotein (36). In poultry, high density lipoprotein extracts cholesterol from tissue cell membranes and esterifies it in the presence of lecithin-cholesterol acyltransferase (37). The newly formed high density lipoprotein is transformed into a mature one by the repeated action of lecithin cholesterol lipid acyltransferase, which in turn transports cholesterol (esters) from senescent cell membranes in peripheral tissues as well as from plasma to the liver for metabolism (38, 39). High density lipoprotein transports cholesterol from extrahepatic tissues to the liver for metabolism (40). In this study, the high density lipoprotein contents in liver and breast muscles were increased, which coincided with a reduction in cholesterol levels, suggesting that guanidinoacetic acid improved lipid metabolism in ducks.

Low density lipoprotein is formed from the metabolites of very low density lipoproteins, which are rich in cholesterol (esters), and apolipoprotein-B, and thus it is the primary form of endogenous cholesterol synthesized by the liver that is translocated to the tissues (41). The adrenal cortex, testis, ovary, and liver tissues have specific low density lipoprotein receptors on their surfaces, which can specifically recognize and bind apolipoproteins on low density lipoprotein particles (42). Low density lipoprotein in plasma binds to the low density lipoprotein receptor on the cell surface to form the low density lipoprotein-receptor complex, which is then ingested into the intracellular endocytic vesicles through endocytosis and then fused with intracellular lysosomes, where the hydrolytic enzymes within the lysosome degrade the low density lipoprotein, with the proteins hydrolyzed to amino acids and cholesterol esters hydrolyzed to cholesterol and fatty acids (43). Free cholesterol can be incorporated into the plasma membrane of poultry cells or esterified and stored as ester-type cholesterol in cells, otherwise metabolized to generate sterol-type actives, and it can also be used to regulate the level of cholesterol in poultry by feedback inhibition of the synthesis of β-hydroxy-β-methylglutaryl monoacyl-CoA reductase (44, 45). In this study, the low density lipoprotein contents in liver and breast muscles were decreased in GA600 and GA800, which was in line with the increase of high density lipoprotein contents and the decrease of cholesterol contents mentioned above and also suggested that guanidinoacetic acid can change the fat metabolism process by regulating the low density lipoprotein contents.

Most of the apolipoproteins (Apo) involved in lipoprotein formation have a bisexual α-helix structure, with uncharged hydrophobic amino acid residues distributed on the non-polar side of the helix and charged hydrophilic amino acid residues distributed on the polar side of the helix, which is conducive to the binding of apolipoproteins to lipids and stabilizes the structure of lipoproteins (46). Different lipoproteins contain different apolipoproteins, and different apolipoproteins have other functions, but their primary function is binding and transporting lipids (46, 47). Apolipoproteins are also involved in biochemical processes such as the regulation of the key enzyme activities in lipoprotein metabolism and lipoprotein receptor recognition, e.g., the tyrosine on the Apo-A1 molecule is an essential motif for binding to high density lipoproteins (46, 48). The Apo-Bs play a role in the reciprocal transfer and exchange of lipoproteins and proteins during lipoprotein metabolism, e.g., cholesterol transport proteins promote the exchange of cholesteryl esters and triacylglycerols among high density lipoprotein, very low density lipoproteins and low density lipoprotein. In contrast, phospholipid transport proteins encourage the interaction of phospholipids between lipoproteins (46, 49). In addition, Apo-B has an irreplaceable role in the biochemical processes of deposition and transport of triglyceride-rich lipids and lipids (50). In this study, the Apo-A1 levels increased, and Apo-B levels decreased in liver and breast muscles in GA600 and GA800, which also echoes the increase in the high-density lipoprotein contents and the decrease in the contents of low-density lipoprotein and cholesterol mentioned above, which also suggested that guanidine acetic acid can change the process of fat metabolism by regulating the apolipoprotein contents.

Lipoprotein lipase, a key enzyme in the classical regulation of lipid metabolism and one of the rate-limiting enzymes of lipid metabolism has an essential effect on fat deposition and intramuscular fat content in animals (51). Lipoprotein lipase catalyzes the breakdown of triglycerides in triglyceride-rich lipoproteins, celiac particles, and very low-density lipoprotein cores into fatty acids and monoglycerides for oxidative energy supply, storage, and utilization by tissues. It plays a vital role in the glucose and lipid metabolism regulation in animals (52). Animal cells generally have no or only a small number of fat droplets; if a more significant number of fat droplets are present within the cells, they are referred to as fat deposits or steatosis, which are formed to a large extent by the buildup of triglycerides within the fat cells leading to cellular hypertrophy (51, 53). Excessive fatty acid intake, fatty acid oxidation, or impaired lipoprotein synthesis can lead to lipoprotein lipase activity changes, ultimately altering poultry lipid metabolism (54, 55). In this study, the lipoprotein lipase levels in liver and breast muscles were increased in GA600 and GA800, which was in line with the reduction of triglycerides mentioned above and also suggested that guanidinoacetic acid can regulate the fat deposition and distribution by regulating lipoprotein lipase. Malate dehydrogenase is an essential enzyme in the tricarboxylic acid cycle that catalyzes the malate dehydrogenation to produce oxaloacetate and NADH^+^ H^+^ (56, 57). This reaction is reversible, but intracellularly produced oxaloacetate is constantly being used to synthesize citric acid, so this reversible reaction tends to proceed more in the direction of oxaloacetate production (58). The fatty acid β-oxidation product acetyl CoA can enter the tricarboxylic acid cycle to generate citric acid for further catabolism, thus indicating that malate dehydrogenase plays a vital role in linking glucose metabolism and fatty acid metabolism in poultry (59, 60). The malate dehydrogenase levels in liver and breast muscles in groups treated with guanidinoacetic acid were decreased in this study, which was in line with the reduction of malondialdehyde, cholesterol, and triglyceride and the increase of phospholipid mentioned above, which also suggested that guanidinoacetic acid can regulate lipid metabolism by regulating malate dehydrogenase.

In poultry, fatty acids or lipoyl CoA activated in the cytosol and the acyl carrier carnitine, also known as *L*-β-hydroxy-gamma-trimethylaminobutyric acid, enter the mitochondria through the mitochondrial membrane catalyzed by carnitine lipoyl transferase (61, 62). Carnitine lipoyl transferase has two isozymes with different antigenic properties, I and II, flanking the outer and inner mitochondrial membrane, respectively (63). Enzyme I, located in the outer mitochondrial membrane, promotes the conversion of fatty acyl groups to lipoyl carnitine, which is transported into the inner membrane through the lipoyl carnitine carrier on the inner mitochondrial membrane and then converted to lipoyl CoA and released carnitine under the enzyme II action (64). Carnitine lipoyl transferase I is the rate-limiting enzyme of the process, which is inhibited by an increase of malonyl CoA in the process of fat synthesis, thus inhibiting fatty acid oxidation (63, 65). Lipoyl CoA entering the mitochondria of poultry cells undergoes dehydrogenation, addition of water, dehydrogenation, and sulfolysis to form lipoyl CoA and acetyl CoA, which have two fewer carbon atoms than the original (66). For saturated fatty acids with an even number of carbon atoms, such as palmitic acid, octadecanestearic acid, and arachidic acid, the final oxidized product is acetyl CoA, which then enters the tricarboxylic acid cycle for further decomposition (67). Unsaturated fatty acids containing double bonds in the mitochondria will go through multiple rounds of β-oxidation of long-chain unsaturated fatty acids under the enoyl CoA isomerase action, and the cis-double bond that may appear between the β and γ carbon atoms will be isomerized to form a trans-double bond between the α and β positions (68).

Similarly, when β-oxidation proceeds to the appearance of a cis-double bond between the γ and δ carbon atoms, a trans-double bond will be formed by the dehydrogenation reaction catalyzed by lipoyl CoA dehydrogenase between the α and β carbon atoms to produce a dienoic acid, followed by the hydrogenation catalyzed by 2,4-diene-lipoyl CoA reductase, which will reduce this dienoic acid to make the trans monoclinic acid, β,γ-enyl lipoyl CoA, which will then be further converted into a trans-diene acid (69). The latter is further isomerized to α,β-enoyl CoA and continues to be oxidatively metabolized to provide energy for poultry (70). In this study, guanidinoacetic acid supplementation did not influence the saturated fatty acid, monounsaturated fatty acid, and polyunsaturated fatty acid contents among groups, which suggested that guanidinoacetic acid has little effect on fatty acid composition in breast muscles and only has a more significant impact on fat deposition and distribution.

Stearoyl CoA desaturase is a critical enzyme in maintaining fat synthesis and energy homeostasis (71). It introduces a double bond between the 9th and 10th carbon atoms of the saturated fatty acids palmitic acid (C16:0) and stearic acid (C18:0), which are converted to the monounsaturated fatty acids palmitoleic acid (C16:1 n-7) and oleic acid (C18:1 n-9), respectively (72). *Free fatty acid receptor 4* is a member of the G-protein-coupled receptor family (GPCRs), which activates various physiological functions such as adipocyte differentiation (73). In this study, the guanidinoacetic acid did not influence the *stearoyl CoA desaturase* and *free fatty acid receptor 4* expressions among groups, which was in line with the reductions in abdominal fat, cholesterol, malondialdehyde, and triglyceride contents, and the insignificant differences in saturated fatty acid, monounsaturated fatty acid, and polyunsaturated fatty acid contents mentioned above. The relative *insulin induced gene 1*, *fatty acid transport protein 1*, and *lipoprotein lipase* expression were increased, the relative *fatty acid synthetase* expression was decreased in groups treated with guanidinoacetic acid, which echoed the increased lipoprotein lipase, total lipase, phospholipids, high density lipoprotein, apolipoprotein-A1 levels, and reduced low density lipoprotein, apolipoprotein-B, abdominal fat, cholesterol, malondialdehyde, and triglycerides levels mentioned above, and suggested that guanidinoacetic acid can modulate the expression of specific genes to regulate fat deposition and distribution in ducks.

## 5 Conclusion

In this study, adding 600 mg/kg dietary guanidinoacetic acid to the diet improved lipid levels, promoted the lipoprotein levels and the lipid metabolism-related gene expressions in ducks.

## Data availability statement

The raw data supporting the conclusions of this article will be made available by the authors, without undue reservation.

## Ethics statement

All procedures involving care and management were approved by the Institutional Animal Care and Use Committee of the Chinese Academy of Tropical Agricultural Sciences (Approval No.: CATAS-20220819-2). The study was conducted in accordance with the local legislation and institutional requirements.

## Author contributions

HW: Formal analysis, Software, Writing – review & editing. JX: Software, Writing – original draft. WP: Formal analysis, Software, Writing – original draft. FJ: Methodology, Software, Writing – original draft. JQ: Methodology, Software, Writing – original draft. QS: Methodology, Software, Writing – review & editing. GH: Methodology, Project administration, Writing – review & editing.
